# Variation between rice accessions in photosynthetic induction in flag leaves and underlying mechanisms

**DOI:** 10.1093/jxb/eraa520

**Published:** 2020-11-07

**Authors:** Liana G Acevedo-Siaca, Robert Coe, W Paul Quick, Stephen P Long

**Affiliations:** 1 Department of Crop Sciences, University of Illinois at Urbana-Champaign, Urbana, IL, USA; 2 Carl R. Woese Institute for Genomic Biology, University of Illinois at Urbana-Champaign, Urbana, IL, USA; 3 High Resolution Plant Phenomics Centre, Commonwealth Scientific and Industrial Research Organization (CSIRO), Plant Industry, Canberra, Australia; 4 C4 Rice Center, International Rice Research Institute, Los Baños, Laguna, Philippines; 5 Department of Animal and Plant Sciences, University of Sheffield, Western Bank, Sheffield, UK; 6 Department of Plant Biology, University of Illinois at Urbana-Champaign, Urbana, IL, USA; 7 Lancaster Environment Centre, Lancaster University, Lancaster, UK

**Keywords:** Atmospheric change, crop improvement, flag leaves, food security, natural variation, photosynthetic induction, rice, rice breeding, Rubisco activation, water use efficiency

## Abstract

Several breeding initiatives have sought to improve flag leaf performance as its health and physiology are closely correlated to rice yield. Previous studies have described natural variation of photosynthesis for flag leaves; however, none has examined their performance under the non-steady-state conditions that prevail in crop fields. Photosynthetic induction is the transient response of photosynthesis to a change from low to high light. Rice flag leaf photosynthesis was measured in both steady- and non-steady-state conditions to characterize natural variation. Between the lowest and highest performing accession, there was a 152% difference for average CO_2_ assimilation during induction (*Ā*_300_), a 77% difference for average intrinsic water use efficiency during induction (iWUE_avg_), and a 185% difference for the speed of induction (IT_50_), indicating plentiful variation. No significant correlation was found between steady- and non-steady-state photosynthetic traits. Additionally, measures of neither steady-state nor non-steady-state photosynthesis of flag leaves correlated with the same measures of leaves in the vegetative growth stage, with the exception of iWUE_avg_. Photosynthetic induction was measured at six [CO_2_], to determine biochemical and diffusive limitations to photosynthesis *in vivo*. Photosynthetic induction in rice flag leaves was limited primarily by biochemistry.

## Teaser

Significant natural variation was found in rice flag leaves for photosynthetic traits measured in steady- and non-steady-state conditions, with implications for improving water use efficiency and photosynthesis in this key crop.

## Introduction

In cereals, the flag leaf is defined as the last leaf to emerge on a mature flowering stem. The flag leaf has a higher photosynthetic capacity relative to lower canopy leaves due to its position at the top of the canopy, which allows for greater interception of light (Adachi *et al.*, 2017). Additionally, high rice yields are closely correlated with the size and health of the flag leaf as they contribute some 50% of assimilates utilized for grain filling (Yoshida, 1981; Ishii, 1993; Nakano *et al.*, 1995; Li *et al.*, 1998). This large contribution of photosynthates is partially due to the proximity of the flag leaf to the grain, as the sink can more easily attract assimilates from closer sources (Sicher, 1993). The rest of the photosynthates used in grain filling are supplied by the leaf immediately below the flag leaf, and by remobilization of stored carbohydrates in leaf sheaths and older senescing leaves (Li *et al.*, 2017; Lin *et al.*, 2018). Agronomic strategies are typically aimed to protect the flag leaf, since its destruction during grain filling is associated with yield losses of up to 45% in rice (Abou-Khalifa *et al.*, 2008).

Consequently, great effort has been dedicated to understanding and optimizing the flag leaf to improve yields (Li *et al.*, 1998). For example, flag leaf size and area can significantly influence grain yield (Li *et al.*, 1998; Zhang *et al.*, 2015). In rice, flag leaves with larger areas are significantly correlated with greater yields and have become a target for breeding programs seeking to achieve an ideal phenotype or ideotype (Zhang *et al.*, 2015). Quantitative trait loci (QTLs) for larger flag leaves have been identified with the purpose of improving yield (Zhang *et al.*, 2015). Additionally, rice yields have been increased by up-regulating *NAL1*, a gene that affects both flag leaf area and photosynthetic rates (Fabre *et al.*, 2016). Increasing rice flag leaf photosynthesis, through either delaying senescence or a more acute leaf angle, has led to higher yields (Mantilla-Perez *et al.*, 2017). Early flag leaf senescence significantly reduces seed-setting rate, 100-grain weight, and yield (Lin *et al.*, 2018). Conversely, delaying senescence prolongs the period in which the flag leaf is photosynthetically active, resulting in more photosynthates that can fill grain (Ishii, 1993; Kobata *et al.*, 2015; Leng *et al.*, 2017). In previous studies, more vertical flag leaf angles resulted in 13% higher photosynthetic rates, reduced photoinhibition, delayed leaf senescence, and 15% higher yields (Chen *et al.*, 2002; Mantilla-Perez *et al.*, 2017).

Open-air elevation of [CO_2_] using free-air CO_2_ enrichment technology is an artificial means of enhancing photosynthesis season-long in field-grown rice and observing the yield response. Modern high-yielding rice lines that have larger single-grain sizes and are able to produce larger panicles show a greater response to this elevation of photosynthesis, in contrast to older lines which appear partially sink limited. The fact that even under these circumstances only 70–80% of spikelets ripen—and less than in the older rice lines—suggests that these high-yielding lines are actually strongly source limited (Hasegawa *et al.*, 2013; Zhu *et al.*, 2015; Sakai *et al.*, 2019; Ainsworth and Long, 2020). These findings suggest that increasing the photosynthetic capacity of the flag leaf would be particularly valuable for these high-yielding cultivars.

Photosynthetic induction is the process by which leaves begin to assimilate CO_2_ upon a transition from low light into high light, and is characterized by a lag in photosynthetic efficiency. Previously, examination of rice leaves in the vegetative growth stage found greater variation for photosynthetic traits during non-steady-state lighting compared with steady state (Acevedo-Siaca *et al.*, 2020). Despite the focus on flag leaf improvement, no studies have characterized the response of flag leaf photosynthesis in non-steady-state conditions or explored the possibility that there is variation in this character that could be utilized for improvement. Recently, it was shown that rice flag leaves are subject to different endogenous aging programs compared with other leaves (Lee *et al.*, 2017). Given its proximity to the major sink in the panicle, feedback would also be expected through non-structural carbohydrate-driven ‘feast’ and ‘famine’ gene expression response networks (Paul *et al.*, 2017). It is therefore likely that photosynthetic properties of flag leaves will differ from those of leaves formed in the vegetative growth stage.

Non-steady-state photosynthesis is important in a field agricultural setting where light is never constant. At the top of the canopy, flag leaves are subject to light fluctuations due to intermittent cloud cover and shadowing by other flag leaves or panicles as wind and sun angles change (Taylor and Long, 2017; Wang *et al.*, 2020). In wheat, time taken for photosynthetic efficiency to recover from transient shadowing over the course of the day in the field was calculated to cost 21% of potential flag leaf assimilation (Taylor and Long, 2017). Improving the rate of recovery in this rarely measured photosynthetic parameter of flag leaves has the potential to increase yield. Limitations to the speed of photosynthetic induction on such shade to sun transitions are due to four main processes: the photoactivation of enzymes involved in the regeneration and production of ribulose-1,5-bisphosphate (RuBP); the buildup of intermediates of carbon metabolism; the activation of Rubisco by Rubisco activase; and stomatal opening (Pearcy *et al.*, 1994; Mott and Woodrow, 2000; Slattery *et al.*, 2018).

This study analyzed the performance of flag leaf photosynthesis in both steady-state and non-steady-state conditions. The objectives were limited to rice flag leaves and aimed to (i) determine the extent of variation between accessions in non-steady-state and steady-state photosynthetic parameters relating to productivity and resource use efficiency across rice accessions; (ii) examine the response of photosynthetic induction of rice flag leaves at different [CO_2_] to understand limitations to induction; and (iii) compare the response of photosynthetic induction in rice flag leaves between accessions and with induction in leaves in the vegetative growth stage of the same accessions as reported in Acevedo-Siaca *et al.* (2020).

## Materials and methods

### Growing conditions and germplasm

Six accessions were selected from the 3000 Rice Genome Project (3K RGP) held at the Germplasm Resources Center at the International Rice Research Institute (IRRI) in Los Baños, Philippines. Accessions were selected on nucleotide mismatches for the gene encoding Rubisco activase, which plays a central role in photosynthetic induction. Seeds were maintained at 50 °C for 1 week to break dormancy and then sown into soil from the IRRI Upland Farm in small pots (4.5 cm diameter×12 cm) and fertilized using 0.4 g^–1^ Osmocote Plus 15-9-12 (The Scotts Company Ltd, Thorne, UK). Seedlings were transferred to larger individual pots (21.5 cm diameter×21.5 cm, 6 liters) after the emergence of the second leaf. These were then placed in a screen house, a type of greenhouse with a glass roof and screen-meshed walls, with no additional lighting or temperature control at IRRI, during the Philippines dry season from March to May 2017. Each pot was kept flooded using a drip irrigation system to simulate paddy conditions (Supplementary Fig. S1).

### Gas exchange measurements

#### Photosynthetic induction

After anthesis, the flag leaf of the main stem was placed in the cuvette of an open gas exchange system (LI-6400XT, LI-COR, Lincoln, NE, USA). Light was provided by an integrated LED head (2×3 LED, LI-6400-02B). Within the cuvette, air temperature was 28 °C to approximate ideal growing conditions, flow rate was 400 µmol s^–1^, and [CO_2_] was maintained at 400 µmol mol^–1^, and water vapor pressure deficit (VPD) at 1.3–1.7 kPa. Prior to measuring photosynthetic induction, rice plants were dark adapted for at least 1 h.

For induction, leaves were first allowed to reach a steady state in low light with a photosynthetically photon flux density (PPFD) of 50 µmol m^–2^ s^–1^ (‘shade’) for 300 s followed by 720 s at 1700 µmol m^–2^ s^–1^ (‘sun’). Gas exchange measures were recorded every 10 s for the duration of the experiment. Measurements were repeated for all six accessions (*n*=8 plants per accession) over 4 d to minimize any age effects. Plants were selected by a randomized design and measured from 08.00 h to 12.00 h, to avoid confounding accessions with any diurnal influences. Net CO_2_ uptake (*A*), stomatal conductance (*g*_s_), intercellular CO_2_ concentration (*C*_i_), transpiration (*E*), and intrinsic water use efficiency (iWUE) were calculated following the equations of Farquhar *et al.* (1981). For a summary of all traits measured, see Table 1.

**Table 1.** A summary of all traits measured and mentioned in the text

Light condition	Trait	Description	Unit
Steady-state	*A*_sat_	Leaf net CO_2_ uptake in saturating light	µmol m^–2^ s^–1^
	*g*_s_	Stomatal conductance	mol m^–2^ s^–1^
	*C*_i_	Intercellular CO_2_ concentration	µmol mol^–1^
	iWUE	Intrinsic water-use efficiency (iWUE=*A/ g*_s_)	µmol CO_2_ mol H_2_O^–1^
	*V*_c, max_	Maximum rate of carboxylation	µmol m^–2^ s^–1^
	J_max_	Maximum rate of electron transport	µmol m^–2^ s^–1^
	CE	Carboxylation efficiency	mol m^–2^ s^–1^
	Γ	Compensation point	µmol m^-2^ s^-1^
	Φ	Quantum yield	Unitless (0–1)
Non-steady-state	*Ā*_300_	Average *A* during first 300 s of induction	µmol m^–2^ s^–1^
	$\bar{g_{s}}$	Average *g*_*s*_ during first 300 s of induction	mol m^–2^ s^–1^
	$\bar{C_{i}}$	Average *C*_*i*_ during first 300 s of induction	µmol mol^–1^
	$\bar{iWUE}$	Average iWUE during first 300 s of induction	µmol CO_2_ mol H_2_O^–1^
	IT_50_	Time to 50% induction	Seconds
	IT_90_	Time to 90% induction	Seconds
	*A*_Max_	Maximum *A* during induction	µmol m^–2^ s^–1^
	*A*_300_	*A* at the end of 300 s	µmol m^–2^ s^–1^
	A*	*A* corrected for stomatal limitation	µmol m^–2^ s^–1^
	1/ τ	Rate constant of Rubisco activation	Seconds
	τ	Time to activation of photosynthesis	Seconds
	*C* Loss	Forgone assimilation	µmol m^–2^

Units and light conditions are included.

Two accessions, previously reported to show very different rates of induction for their leaves during the vegetative phase of growth, IR64-21 and AUS 278, were selected for further analysis of flag leaf induction at different [CO_2_] (Acevedo-Siaca *et al.*, 2020). Induction was measured following the protocol described above for induction, but at a cuvette [CO_2_] of either 100, 200, 300, 400, 600, or 800 µmol mol^–1^ during induction. The order of cuvette [CO_2_] treatments for each individual leaf was randomized to avoid confounding [CO_2_] with time. Leaves were dark adapted for a minimum of 1 h between measurements at the different [CO_2_] at which they would later be measured. To determine limitations through induction, *A* was plotted against *C*_i_ for different time points, following the procedure of Soleh *et al.* (2016) and of Acevedo-Siaca *et al.* (2020). Five time points were selected for further analysis: 60, 180, 300, 360, and 700 s from the initiation of induction by transfer from darkness to high light (1700 µmol m^–2^ s^–1^). This allowed determination, at each time point, as to whether photosynthesis within the mesophyll was limited by the apparent maximum rates of RuBP regeneration (*J*_max_) or RuBP carboxylation by Rubisco (*V*_c,max_).

#### Steady-state measurements

For steady-state measurements, flag leaves were allowed to reach constant rates of *A* and stomatal conductance (*g*_s_) at 1700 µmol m^–2^ s^–1^ PPFD. Cuvette conditions for steady-state measurements were as described above for photosynthetic induction.

### Calculations

#### The rate constant of Rubisco activation, time to activation of photosynthesis, and forgone assimilation

The rate constant of Rubisco activation (1/τ) was calculated by fitting the slope of the linear phase of the natural log of corrected photosynthetic induction [ln(*A*_f_*–A*)] as described in Woodrow and Mott (1989). A* is photosynthesis at a point in time during induction corrected to a *C*_i_ value of 300 µmol mol^–1^ to remove limitation from stomata. *A*_f_* is the corrected value for photosynthesis at a *C*_i_ of 300 µmol mol^–1^ at the end of the induction. The correction to *C*_i_ was made following the methods of Soleh *et al.* (2016):

$$\begin{array}{l} A^{⋆}=A\times \frac{300}{C_{i}} \end{array}$$ (1)

The time required to complete the activation of photosynthesis (τ) was calculated by taking the reciprocal of the rate constant of Rubisco activation (Woodrow and Mott, 1989).

The integrated amount of CO_2_ uptake foregone due to the lower rates through induction compared with steady-state (*C* Loss_t_) was calculated as in Acevedo-Siaca *et al.* (2020):

$$\begin{array}{l} C\;Loss_{t}=\left(A-{\bar{A}}_{t}\right)\times t \end{array}$$ (2)

where *A* is the steady-state rate of uptake and *Ā*_*t*_ the average rate across the measured time period from the start of the induction (*t*), 300 s and 700 s, respectively.

### Statistical analyses

All statistical analyses and model fitting used R (version 3.5.2, R-project) (R Core Team, 2020). Normal distribution and homogeneity of variances were tested by the Shapiro–Wilk test and Brown–Forsythe test, respectively. Assumptions were met and ANOVA was performed followed by Tukey’s mean discrimination analysis, using the R-Project: ‘agricolae: Statistical Procedures for Agricultural Research’ package. Pearson correlation coefficients between different photosynthetic measures were calculated using accession mean values (R; ‘corrplot’ and ‘Hmisc’).

## Results

### Characterizing photosynthesis in flag leaves

**Fig. 1.**
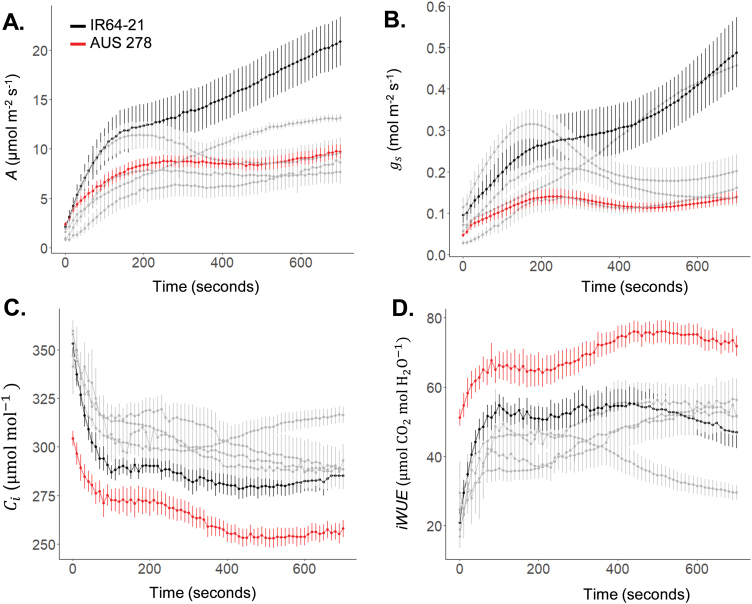
The response of flag leaves from six rice accessions during photosynthetic induction. (A) Net leaf CO_2_ assimilation (*A*), (B) stomatal conductance (*g*_s_), (C) intercellular CO_2_ concentration (*C*_i_), and (D) intrinsic water use efficiency (iWUE=*A*/*g*_s_) with time (*t*) of induction upon change at 0 s from low light to high light (50 µmol m^–2^ s^–1^ to 1700 µmol m^–2^ s^–1^). The measurement was taken at an ambient [CO_2_] of 400 µmol mol^–1^. Two accessions, AUS 278 (red) and IR64-21 (black), were selected for further study at varied [CO_2_]. The other four accessions are Dechangbyeo, Fei Zhao 12, M 102, and Malogbana. Each point is the mean ±SE) of eight plants (*n*=8).

**Fig. 2.**
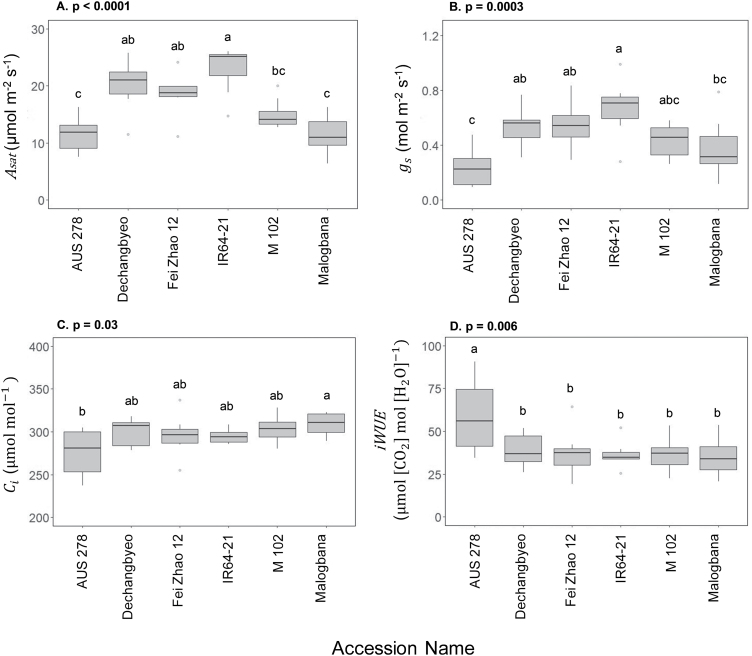
Mean and variation for flag leaf steady-state photosynthetic performance in six rice accessions. (A) Leaf CO_2_ assimilation (*A*), (B) stomatal conductance (*g*_s_), (C) intrinsic water use efficiency (iWUE=*A/g*_s_), and (D) intercellular CO_2_ concentration (*C*_i_). Accessions are ordered by median performance. Letters are indicative of a significant difference between accessions.

**Fig. 3.**
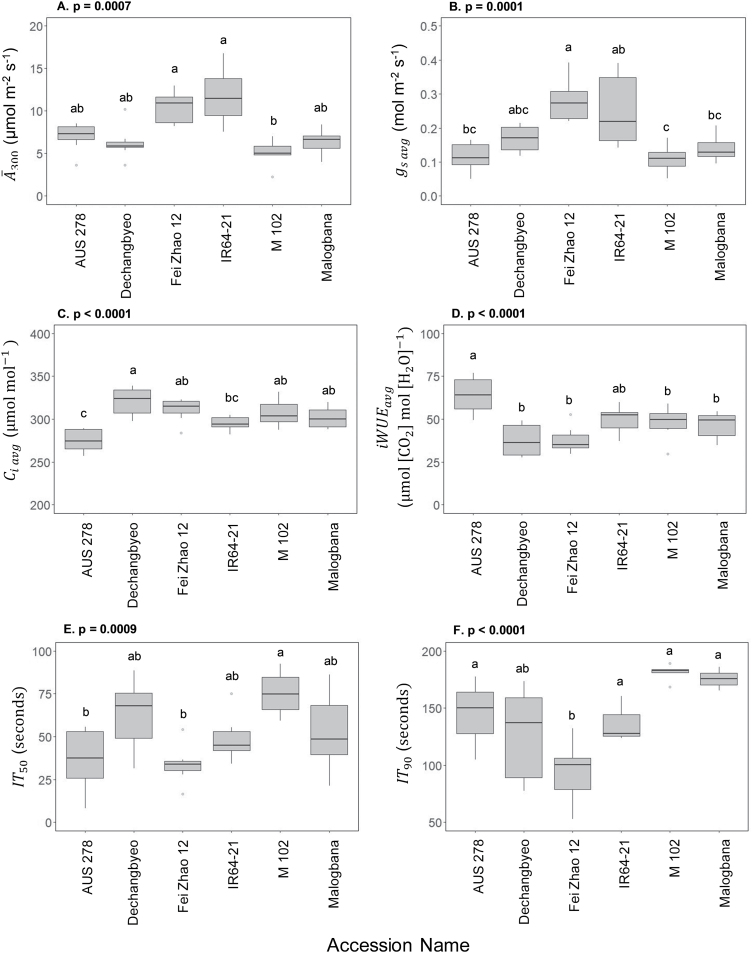
Mean and variation for flag leaf non-steady-state photosynthetic performance in six rice accessions. (A) CO_2_ assimilation during the first 300 s of induction (*Ā*_300_), (B) average stomatal conductance during the first 300 s of induction (*g*_s avg_), (C) average intrinsic water use efficiency (iWUE_avg_=*Ā*_300_/*g*_s avg_), (D) average intercellular CO_2_ concentration (*C*_i avg_), (E and F) time at which *A* reached 50% and 90% of *A*_300_ (IT_50_ and IT_90_, respectively). Accessions are ordered by median performance. Letters are indicative of a significant difference between accessions.

**Fig. 4.**
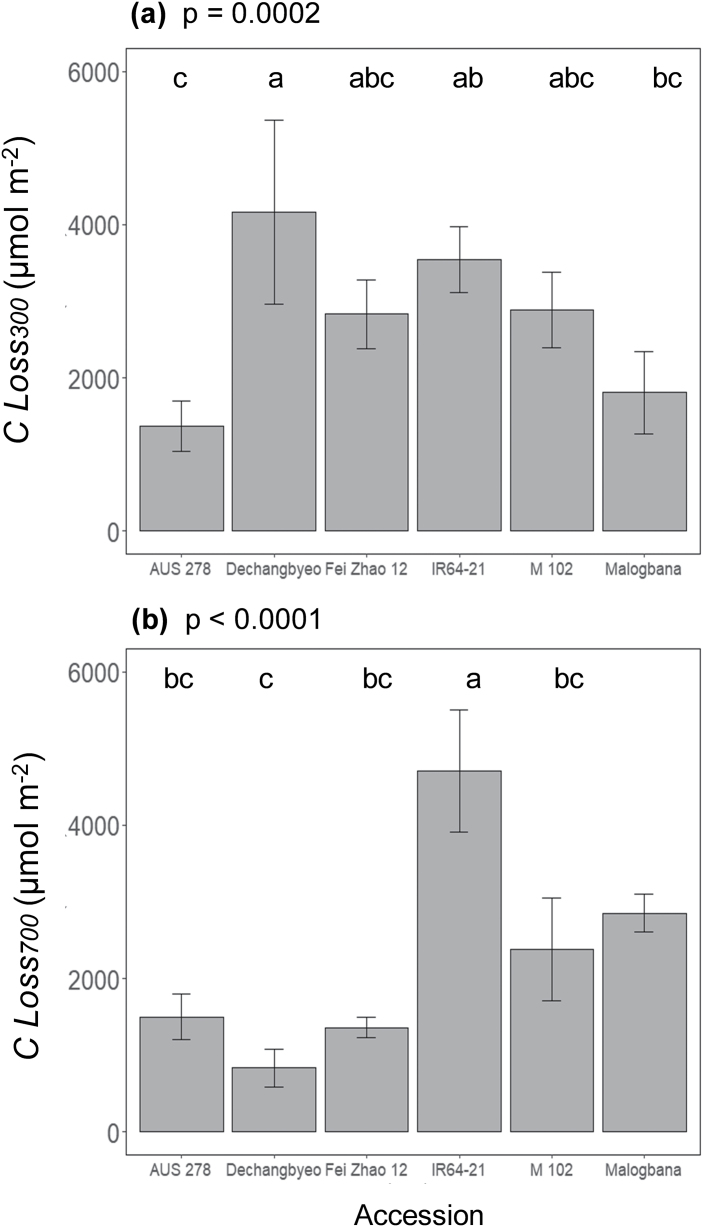
The integra1 of CO_2_ uptake forgone due to the lower than steady-state rates through the first (A) 300 s and (B) 700 s of induction compared with steady state (*C* Loss_300_ and *C* Loss_700_, respectively).

Significant variation was found across accessions for photosynthetic traits in both non-steady-state and steady-state conditions (Figs 1, 2). Significant differences were seen in steady-state parameters such as CO_2_ uptake in saturating light (*A*_sat_, *P*<0.0001), stomatal conductance (*g*_s_, *P*=0.0003), intercellular CO_2_ concentration (*C*_i_, *P*=0.03), and intrinsic water use efficiency (iWUE, *P*=0.006) (Fig. 2). Between the highest and lowest performing accessions, during induction there was a 152% difference for average CO_2_ assimilation (*Ā*_300_) (M 102, 4.1 µmol m^–2^ s^–1^ versus IR64-21, 10.4 µmol m^–2^ s^–1^), 77% difference for average iWUE (iWUE_avg_) (Fei Zhao 12, 36.2 µmol CO_2_ mol H_2_O^–1^; AUS 278, 64.1 µmol CO_2_ mol H_2_O^–1^), and a 185% difference for the time to 50% induction (IT_50_) (Fei Zhao 12, 34.3 s; M 102, 98.2 s) (Fig. 3). Significant differences were also found among accessions for *C* Loss_300_ and *C* Loss_700_ (Fig. 4).

### Understanding biochemical limitations to photosynthetic induction in flag leaves

**Fig. 5.**
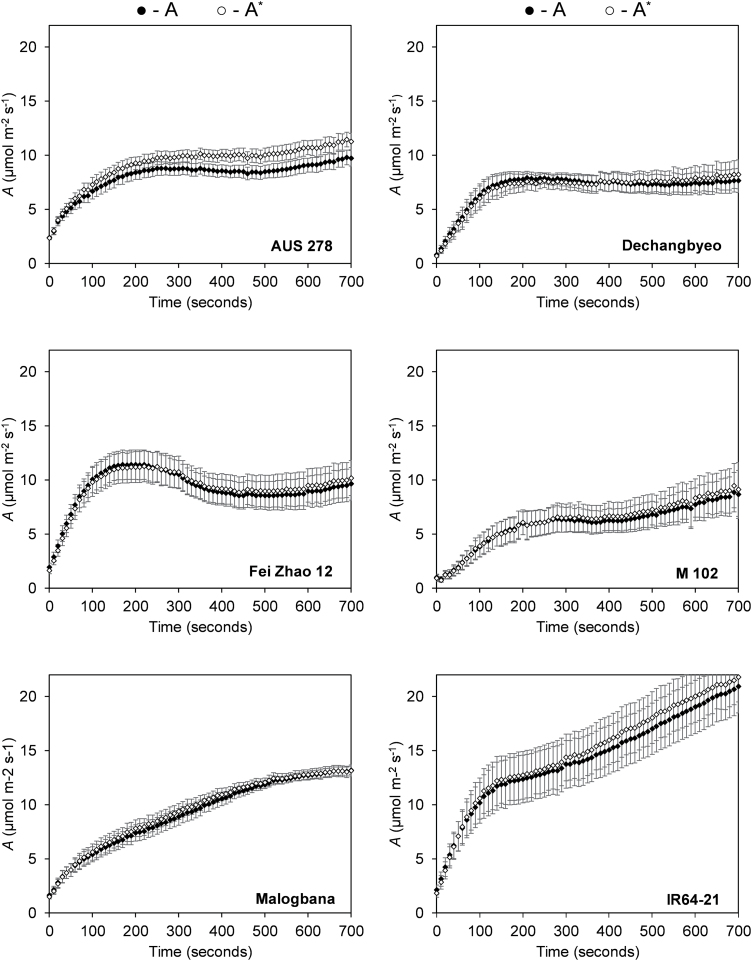
The response of uncorrected leaf CO_2_ assimilation (*A*; filled circles) and the response of leaf CO_2_ assimilation corrected for stomatal limitation (A*; open circles) over time in flag leaves of six rice accessions. The first line at 100 s indicates the mean time for the activation of Rubisco (τ) per accession. Each point represents the mean of at least six plants ±SE (*n*=6–8).

Photosynthetic induction in flag leaves was limited primarily by biochemistry, rather than stomata, during the first 300 s of induction (Fig. 5). The rate of photosynthesis corrected to remove stomatal limitation (A*) did not differ significantly from the uncorrected *A* values, suggesting that biochemical limitation dominated for these accessions (Fig. 5). This was also evident by the fact that *C*_i_ was higher than at steady state through most of the induction (Fig. 1C). A* showed only small differences from the corresponding *A* (~3–4%), except for AUS 278 which had an average difference of ~13% between *A* and A* (Fig. 5).

**Fig. 6.**
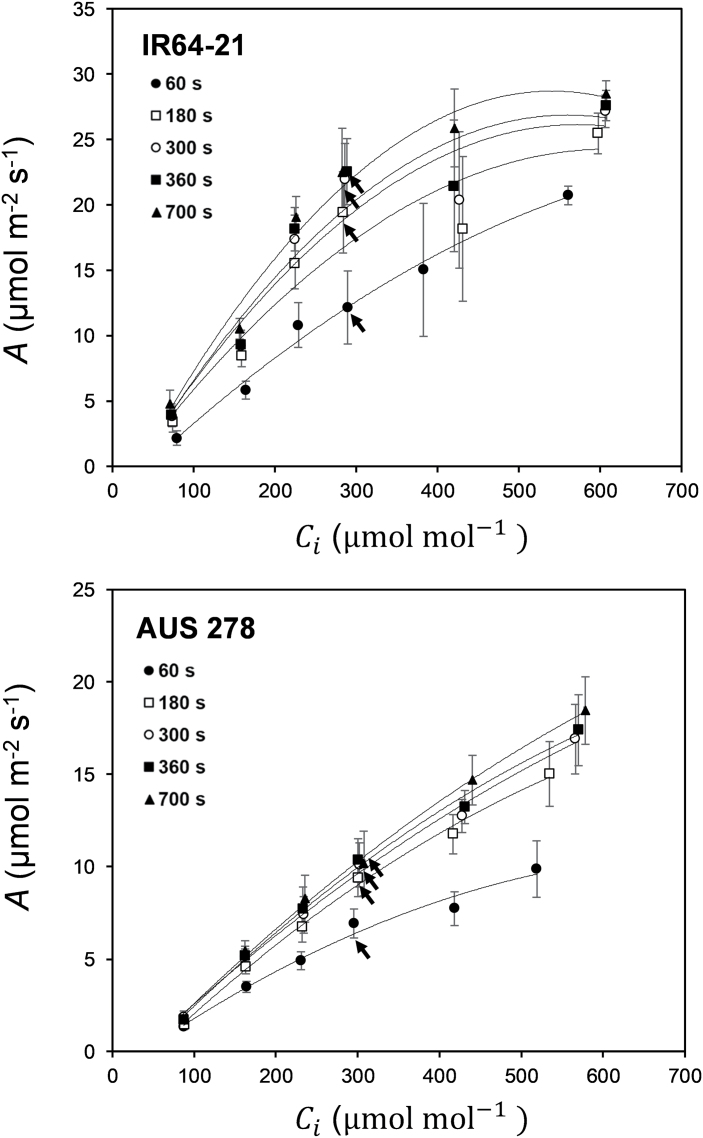
The responses of leaf CO_2_ assimilation (*A*) to intercellular [CO_2_] (*C*_i_) at different points in time after the beginning of photosynthetic induction for IR64-21 and AUS 278. Times after induction were: 60 s (filled circles), 180 s (open squares), 300 s (open circles), 360 s (filled squares), and 700 s (filled triangles) from the start of induction. The operating point of each curve at 400 µmol mol^–1^ atmospheric [CO_2_] (*C*_a_) is indicated with a black arrow. Each point is the mean (±SE) of four plants of each rice accession.

Photosynthetic induction was measured at six different [CO_2_] values in two selected accessions, AUS 278 and IR64-21. As expected, as [CO_2_] increased, *A* increased in both accessions (Supplementary Fig. S3). Also as expected, stomatal conductance decreased with increased [CO_2_] (Supplementary Fig. S3). As time after the beginning of induction increased, the amount of CO_2_ assimilated increased as well (Fig. 6). This response to increasing CO_2_ assimilation over time was seen in both selected accessions (Fig. 6).

Utilizing these data, the operating point at an ambient [CO_2_] (*C*_a_) of 400 µmol mol^–1^ was calculated for each curve. The operating point fell on the initial slope of the *A/C*_i_ curve, indicating limitation by Rubisco for both accessions at all time points (Fig. 6). Photosynthesis was corrected for stomatal limitation at the different [CO_2_] (Supplementary Figs S4, S5) to calculate the rate constant for Rubisco activation, τ, and forgone assimilation (*F*). The rate constant for Rubisco activation and τ was not significantly influenced by [CO_2_] (Supplementary Figs S6, S7) while *C* Loss_300_ increased significantly with [CO_2_] (Supplementary Fig. S7).

Finally, the photosynthetic response curves to PPFD, measured at low [CO_2_] (≤300 µmol mol^–1^), were used to infer the response of *V*_c,max_ to a PPFD *in vivo*. These results indicated a significant difference between AUS 278 and IR64-21, with AUS 278 being more strongly limited by the rate of carboxylation (Supplementary Fig. S8). This contrasts with induction at ambient [CO_2_], where AUS 278 and IR64-21 did not vary significantly in photosynthetic induction traits *Ā*_300_, *g*_s avg_, *C*_i avg_, iWUE_avg_, IT_50_, and IT_90_ (Fig. 3).

### Comparing steady- and non-steady-state photosynthetic performance

**Fig. 7.**
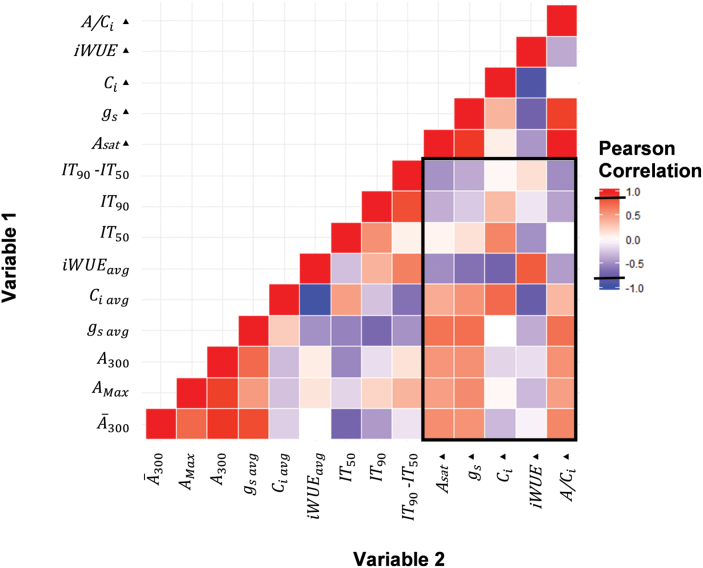
Pearson correlation (*R*) of all measured dynamic and steady-state (filled tiangles) photosynthetic traits measured in rice flag leaves. Negative correlations are in blue, positive correlations are in red. Traits at steady-state are: intrinsic water-use efficiency (iWUE=*A/g*_s_), transpiration (*E*), intercellular CO_2_ concentration (*C*_i_), stomatal conductance (*g*_s_), and net CO_2_ assimilation in saturating light (*A*_sat_). Traits at non-steady state over the first 300 s of induction are: the time at which *A* reached 50% and 90% of *A*_300_ (IT_50_ and IT_90_, respectively), average *C*_i_ during first 300 s of induction (*C*_i avg_), average intrinsic water use efficiency (iWUE_avg_=*Ā*_300_/*g*_s avg_), average *g*_s_, the maximum *A* during induction (*A*_Max_), *A* at the end of this period (*A*_300_), and the average *A* (*Ā*_300_). A significant *R* value is marked by a black line on the scale (0.8).

The photosynthetic performance of accessions in non-steady-state and steady-state conditions was compared using Pearson’s correlation coefficient. Significant (*P*<0.05) correlations were found between the different photosynthetic traits measured in steady-state and between the different traits measured in non-steady-state conditions (Fig. 7). However, there were no significant correlations between traits measured at steady state and their corresponding traits measured at non-steady state (Fig. 7). Additionally, as was previously found in vegetative-phase leaves, there was a significant correlation (*P*<0.05) between *Ā*_300_ and time at which *A* reached 50% induction (IT_50_) (Fig. 7).

### Comparing photosynthetic induction performance between flag leaves and vegetative-phase leaves

**Fig. 8.**
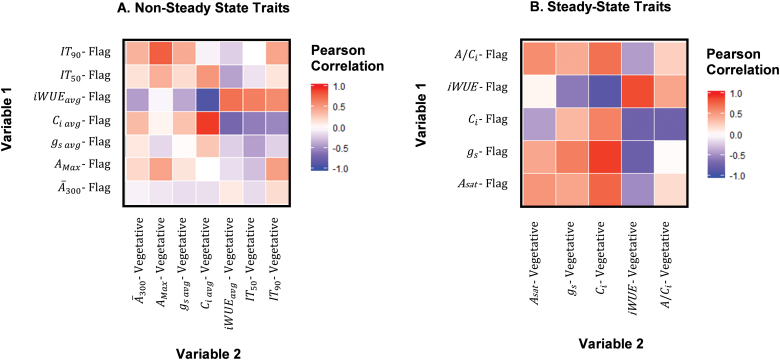
Pearson correlation analysis between photosynthetic traits measured in flag leaves for the six cultivars measured here with the values obtained on leaves in the vegetative growth stage for the same cultivars in a previous study (Acevedo-Siaca *et al.* 2020). (A) Non-steady state; (B) steady state. Traits are as defined in Fig. 3.

The photosynthetic traits measured here for flag leaves were compared with those made for leaves in the vegetative growth stage of the same accessions in a previous study (Acevedo-Siaca *et al.*, 2020). Significant correlations were found between steady-state *C*_i_ in leaves in the vegetative growth stage and iWUE in flag leaves, *C*_i_ in leaves in the vegetative growth stage and *g*_s_ in flag leaves, and iWUE in leaves in the vegetative growth phase and flag leaves (Fig. 8A). In non-steady-state conditions, significant correlations were found between *C*_i avg_ in leaves in the vegetative growth stage and both *C*_i avg_ and iWUE_avg_ in flag leaves (Fig. 8). However, there was no significant correlation between any measure of CO_2_ assimilation between flag leaves and leaves during the vegetative growth phase.

## Discussion

Currently, no study has aimed to characterize the photosynthetic induction response in rice flag leaves. With respect to the objectives as outlined in the Introduction, the following key answers were obtained. (i) There were substantial differences among the six accessions of ~150% between leaf CO_2_ uptake over the course of induction, with smaller differences in the light-saturated steady state (Figs 2, 3). This suggests significant scope for exploiting germplasm to increase rice photosynthesis in this key leaf for grain filling. (ii) Analysis of the responses of CO_2_ uptake to intercellular CO_2_ concentration showed that the *in vivo* activity of Rubisco (*V*_c,max_) limited photosynthesis throughout induction (Fig. 6), suggesting that breeding or bioengineering increased Rubisco content and activity as a means to increase rice productivity. (iii) When the results obtained here for flag leaves were compared with those for the same accessions in the earlier study of leaves in the vegetative growth stage (Acevedo-Siaca *et al.*, 2020), there was little correspondence between photosynthetic parameters for the two growth stages, with the exception of iWUE (Fig. 8). This suggests that breeding efforts to improve crop photosynthesis would need to address selection at both growth stages, although improved WUE could be selected at the vegetative stage alone, making this more tractable. The following discussion places these new findings in the context of prior studies and potential for increasing rice photosynthetic capacity, efficiency of resource use, and productivity.

### Significant variation for photosynthetic traits in rice flag leaves

Previously, it was suggested that natural variation for photosynthesis was more prevalent in the flag leaves than for leaves in the vegetative growth stage of cereal crops, a finding confirmed here for rice (Dunstone *et al.*, 1973; Bansal *et al.*, 1993). Previous studies have focused on either steady-state or more recently non-steady-state photosynthesis, and demonstrated diversity within crop germplams (Flood *et al.*, 2011; Gu *et al.*, 2012; Driever *et al.*, 2014; Soleh *et al.*, 2016, 2017; Acevedo-Siaca *et al.*, 2020; De Souza *et al.*, 2020; Taniyoshi *et al.*, 2020). Here, we found that significant natural variation exists for photosynthetic traits in both steady- and non-steady-state conditions (*A* 6.4–25.9 µmol m^–2^ s^–1^; *Ā*_300_ 2.3–16.7 µmol m^–2^ s^–1^) in the flag leaf. Indeed, 43, 12, and 68% more variation was found here between flag leaves when compared with leaves in the vegetative growth stage of the same accessions (Acevedo-Siaca *et al.*, 2020) for *Ā*_300_, iWUE_avg_, and IT_50_, respectively. This is significant given the key role of the flag leaf.

### Further evidence for the lack of correlation between steady- and non-steady-state measurements

Consistent with studies of soybean, cowpea, and leaves in the vegetative growth stage of rice, no significant correlations were found between parameters of photosynthetic CO_2_ uptake in steady- and non-steady-state light in rice flag leaves (Soleh *et al.*, 2016; Acevedo-Siaca *et al.*, 2020; De Souza *et al.*, 2020). These studies, together with the present study, provide compelling evidence for a reconsideration of when during crop development we measure and how we measure photosynthesis when considering crop improvement. In particular, clear evidence that steady-state measurements do not indicate photosynthetic efficiency under the non-steady-state fluctuating light conditions that can be dominant in the field should be noted. However, the greater between-accession variability of non-steady-state photosynthesis highlights a greater opportunity for increasing net crop CO_2_ uptake that could meet the apparent strong source limitation of modern high-yielding cultivars (Hasegawa *et al.*, 2013; Zhu *et al.*, 2015; Sakai *et al.*, 2019).

It is possible that a balance for the distribution of resources between photosynthetic proteins underlies the differences between steady-state and non-steady-state photosynthesis. Previous studies have suggested a trade-off between maximum rates of photosynthesis in steady-state saturating light versus the speed of induction due to limited amounts of nitrogen invested and divided between Rubisco and Rubisco activase content (Woodrow and Mott, 1989; Mott and Woodrow, 2000). It is hypothesized that plants grown in fluctuating light environments that do not experience steady-state conditions frequently would benefit from having higher Rubisco activase content to be able to respond more quickly to changes in light (Mott and Woodrow, 2000; Yamori *et al.*, 2012; Carmo-Silva and Salvucci, 2013; Kaiser *et al.*, 2016). However, leaves that experience fewer sunflecks and have more exposure to direct sunlight would benefit from investing a higher proportion of resources in Rubisco to sustain higher photosynthetic rates at steady state (Mott and Woodrow, 2000). Such a trade-off is supported by the finding that antisense down-regulation of Rubisco activase increased Rubisco content in rice leaves (Jin *et al.*, 2006). This trade-off between Rubisco and Rubisco activase content could help to partially explain the lack of correlation between steady- and non-steady-state photosynthesis, as an increase in the protein that helps the leaf excel in induction acts to the detriment of the protein needed at steady state. The trade-off between steady- and non-steady-state photosynthesis was shown clearly in IR64-21, which had the highest steady-state *A*_sat_ yet was among the slowest to reach 90% of its steady-state level (IT_90 A_) during induction (Figs 2, 3).

### Photosynthetic induction is primarily limited by biochemistry in flag leaves

At ambient [CO_2_], rice flag leaves are predominantly limited by biochemistry, specifically the maximum activity of Rubisco (Figs 5, 6). Differences between *A* and A* at ambient [CO_2_] were generally small, indicating little limitation by stomata (Fig. 5). This is likely to be due to the shape and small size of rice stomata allowing fast responses (McAusland *et al.*, 2016), but also the evolutionary history of rice, which was domesticated from emergent aquatic progenitors and then bred in paddy conditions where water would not be limiting to the plant (Nay-Htoon *et al.*, 2018).

Calculation of the operating point at ambient [CO_2_] (*C*_a_=400 µmol mol^–1^) in AUS 278 and IR64-21 showed that photosynthesis during induction was predominantly limited by Rubisco, and not affected by either the capacity for regeneration of RuBP or triose phosphate utilization, throughout the induction and into steady state. The operating point is on the initial slope of the *A/C*_i_ curve, throughout (Fig. 6). This parallels the previous findings for rice leaves in the vegetative growth stage (Acevedo-Siaca *et al.*, 2020) and suggests that the predominant limitation to photosynthetic CO_2_ assimilation at steady state and non-steady state throughout the life cycle of rice is consistently due to the *in vivo* capacity and, presumably, amount of Rubisco. This is also consistent with the recent observation that transgenic up-regulation of Rubisco in rice significantly increases paddy yield (Long, 2020; Yoon *et al.*, 2020). One caveat is that this analysis is based on *C*_i_ and not the CO_2_ concentration at Rubisco (*C*_c_). Ease of movement of CO_2_ from the intercellular space to Rubisco is governed by mesophyll conductance (*g*_m_), which was not measured here. So, it is possible that activation of *g*_m_, as well as Rubisco, could be a limiting factor. However, prior work with other species has suggested that activation of *g*_m_ is likely to be faster than activation of Rubisco (Deans *et al.*, 2019).

### Photosynthetic performance shows little correlation between flag and vegetative-phase leaves

The only significant correlations found between flag and leaves in the vegetative growth stage were for *C*_i_ and iWUE (Fig. 8). These results suggest that iWUE is not be affected by rice developmental stage and could be consistent throughout the lifetime of these rice plants. Water availability is the biggest limitation to agricultural production worldwide and is expected to pose an even greater limitation with climate change and population growth (Wassmann *et al.*, 2009; Ort and Long, 2014; Oladosu *et al.*, 2019). If iWUE is consistent throughout the life cycle of rice, it could allow breeders to screen for high iWUE early in development, saving time and resources in selecting germplasm in breeding more water use-efficient plants.

Otherwise, no significant correlations were found between the photosynthetic performance of flag leaves and leaves in the vegetative growth stage in both steady- and non-steady-state conditions (Fig. 7). For example, elite cultivar IR64-21 was outperformed by other accessions in measurements of photosynthesis in leaves in the vegetative growth stage during steady- and non-steady-state conditions (Acevedo-Siaca *et al.*, 2020). However, IR64-21 flag leaves had the highest photosynthetic rates during photosynthetic induction and at steady state, significantly outperforming the other accessions (Figs 1, 2; Supplementary Fig. S3). This suggests that increased flag leaf CO_2_ uptake may have been inadvertently improved through conventional breeding selection. This corresponds with other evidence that flag leaf photosynthesis has been improved unintentionally through breeding for higher yield potential. Newer and higher yielding rice varieties have flag leaves with higher photosynthetic rates per unit area than older varieties (Ishii, 1993). Additionally, flag leaves in *Oryza sativa* were found to maintain higher photosynthetic rates for longer relative to wild *Oryza* species (Ishii, 1993), which is curiously in contrast to wheat (Dunstone *et al.* 1973). These studies suggest that deliberate breeding for increased flag leaf photosynthesis might be a fertile avenue for a further increase in rice yield potential.

However, while an increased emphasis on flag leaf photosynthesis can lead to higher yields (Fabre *et al.*, 2016), there should still be a focus on improving photosynthetic efficiency throughout the life cycle. Photosynthesis in leaves during the vegetative stage is important in establishing the plant and developing a robust root system and tillers capable of becoming reproductive. Improved photosynthesis in leaves in the vegetative growth stage results in increases in non-storage carbohydrates in leaves and stems, which can subsequently be remobilized, for grain filling. The expected ideotype would therefore be an accession which shows high capacity and efficiency during both growth phases.

### Non-steady-state photosynthesis—a practical target for breeding?

Non-steady-state photosynthesis has received little attention in breeding, largely because the gas exchange methods required to effectively phenotype traits are not practical for large-scale testing. However, it was recently shown in wheat that large-scale screening of non-steady-state photosynthesis could be achieved effectively with excised leaves using modulated chlorophyll fluorescence imaging (McAusland *et al.*, 2019). This high-throughput technique could make selection and breeding for improved efficiency under non-steady-state conditions practical, and probably more effective than selecting for improvement under steady-state conditions. Additionally, these methods could allow for the improvement of iWUE under non-steady-state conditions. As noted above, current methods of breeding paddy rice for yield may have lowered WUE. This study has revealed considerable variation—even within the limited germplasm examined—and a correlation in WUE between vegetative and reproductive growth. This finding suggests that selection of improved WUE could be achieved by screening during early growth. Furthermore, the development of integrated thermal and modulated fluorescence imaging of instantaneous WUE would now allow high-throughput phenotyping of this trait (McAusland *et al.*, 2013). Accelerated breeding of rice lines requiring less water would help address the rising pressures on water supplies in many paddy rice-growing regions (Schyns *et al.*, 2019). These benefits that go beyond increased yield are imperative for creating more sustainable agricultural systems that utilize this planet’s limited resources with more discretion, especially in the face of global climate change and a growing human population.

## Supplementary data

The following supplementary data are available at *JXB* online.

Fig. S1. Growing conditions at the IRRI used in the study.

Fig. S2. Corrected photosynthesis for time to activation of photosynthesis (τ).

Fig. S3. Induction of leaf CO_2_ uptake (*A*) and stomatal conductance (*g*_s_) at six [CO_2_] in IR64-21 and AUS 278.

Fig. S4. Leaf CO_2_ uptake (*A*) and leaf CO_2_ uptake corrected for stomatal limitation (A*) in AUS 278.

Fig. S5. Leaf CO_2_ uptake (*A*) and leaf CO_2_ uptake corrected for stomatal limitation (A*) in IR64-21.

Fig. S6. Corrected photosynthesis for time to activation of photosynthesis (τ) in AUS 278 and IR64-21.

Fig. S7. Rate constant of Rubisco activation (1/τ), time to activation of photosynthesis (τ), and forgone assimilation in six [CO_2_] in AUS 278 and IR64-21.

Fig. S8. *V*_c,max_ during photosynthetic induction in AUS 278 and IR64-21.

Table S1. Description of accessions used in this study.

eraa520_suppl_Supplementary-Figures-S1-S8_and_Table-S1

## Data Availability

The data that support the findings of this study are openly available in the University of Illinois Data Bank at https://doi.org/10.13012/B2IDB-3596430_V1 (Acevedo-Siaca, Liana; Long, Stephen (2020): Photosynthetic induction of rice flag leaves. University of Illinois at Urbana-Champaign.)
