# Supplementary material for: Variation between rice accessions in photosynthetic induction in flag leaves and underlying mechanisms
Source: J Exp Bot. 2020 Nov 7;72(4):1282–94. doi: 10.1093/jxb/eraa520 (PMC7904153; doi:10.1093/jxb/eraa520)
Supplement: eraa520_suppl_Supplementary-Figures-S1-S8_and_Table-S1 [file eraa520_suppl_supplementary-figures-s1-s8_and_table-s1.pdf]

Journal of Experimental Botany Supplementary Materials Figs S1-S8 and Table S1

Article Title: Variation between rice accessions in photosynthetic induction in flag leaves and underlying mechanisms.

Authors: Liana Acevedo-Siaca, Robert Coe, W. Paul Quick, Stephen P. Long

SUPPLEMENTARY FIGURES AND TABLE:

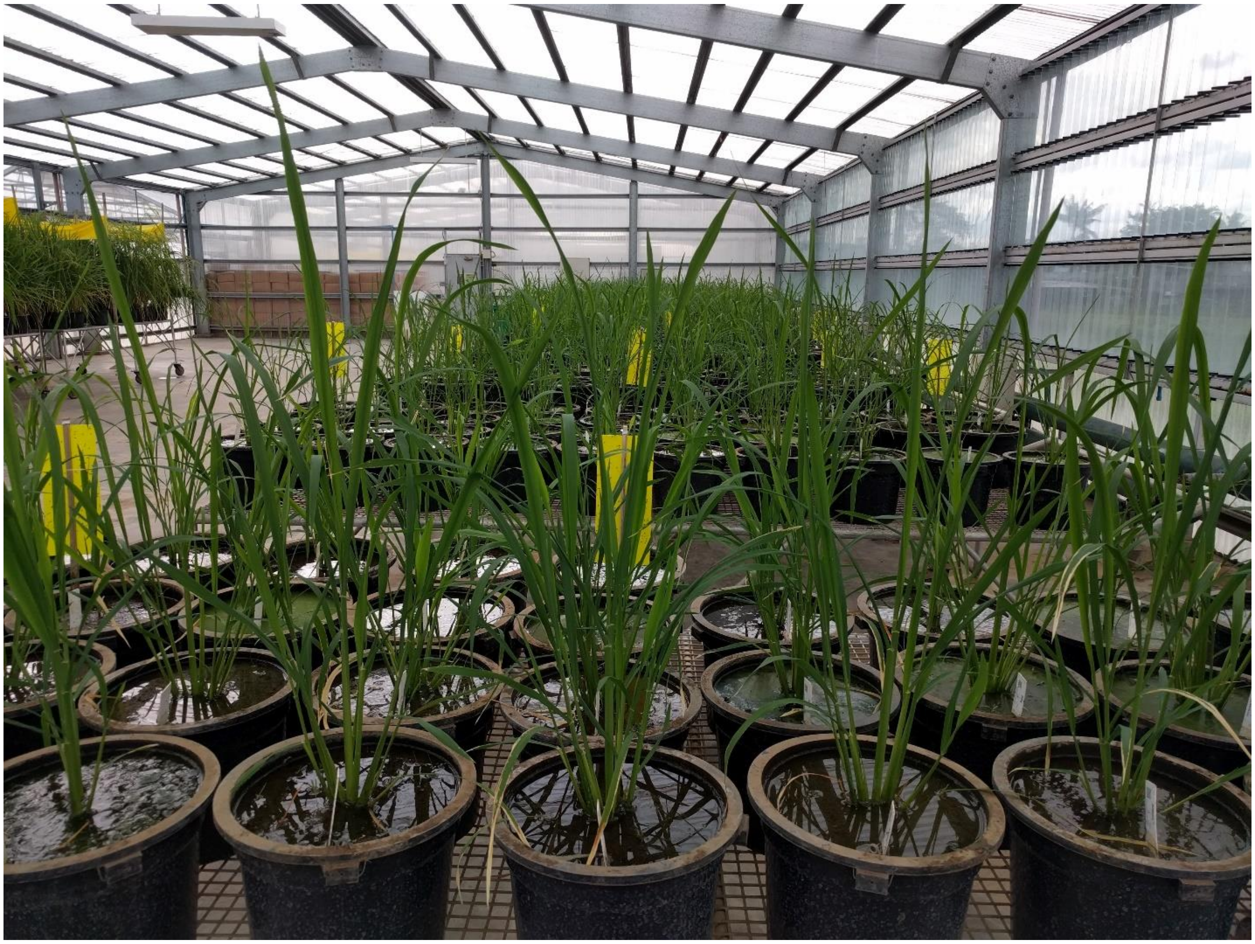

Fig. S1. Growing conditions of rice accession at the International Rice Research Institute (IRRI) in Los Baños, Philippines.

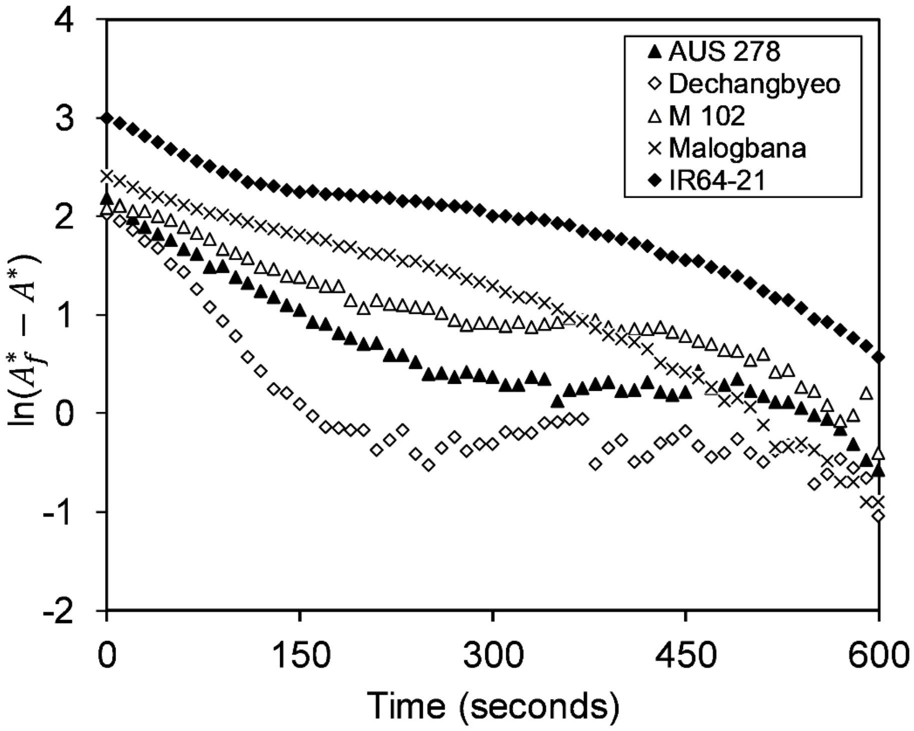

Fig. S2. The natural log of (A_f^*-A^*) over time, where A_f^* is CO_2 assimilation corrected for stomatal limitation at the end of photosynthetic induction and A^* is corrected CO_2 assimilation each time point (*t*) during induction. Each point illustrated is the mean of at least 6 plants (n = 6-8).

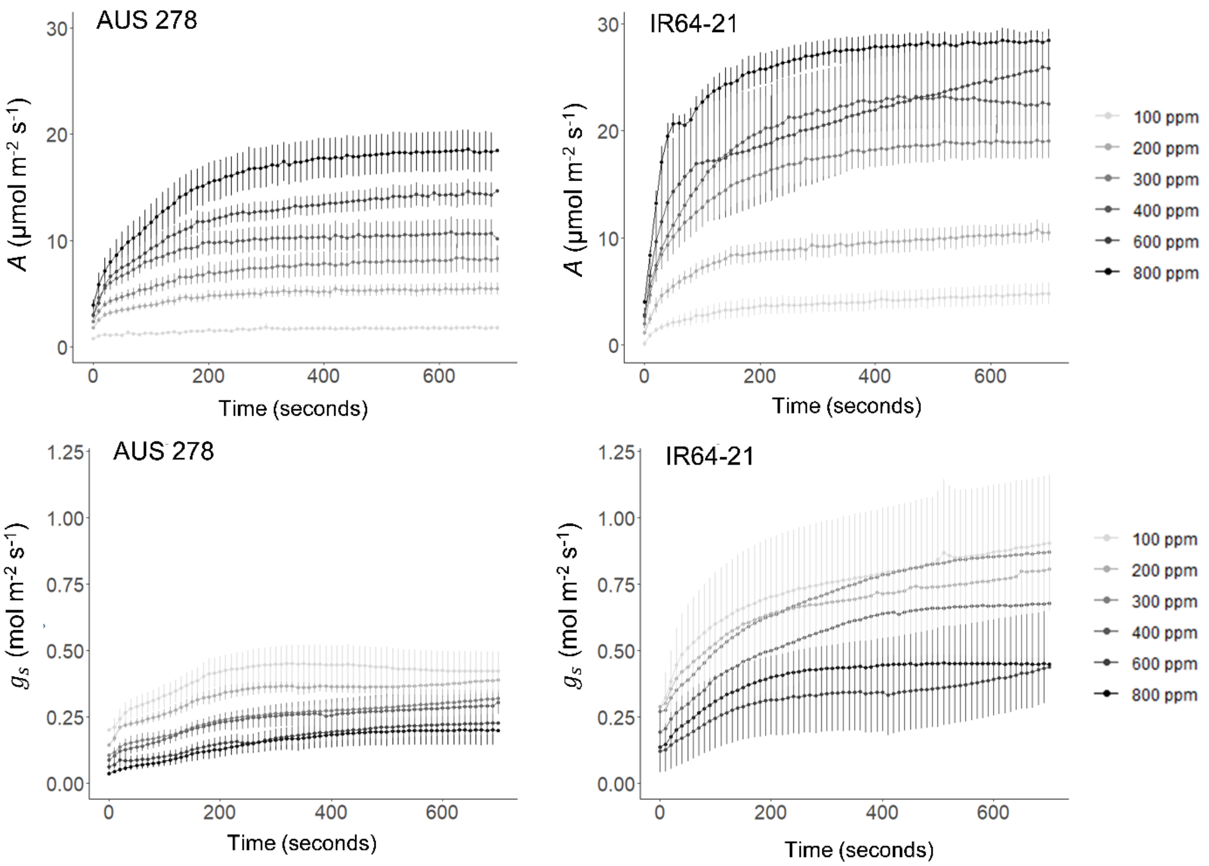

Fig. S3. Induction of leaf CO_2 assimilation (*A*) and stomatal conductance (g_s) at [CO_2]: 100, 200, 300, 400, 600, and 800 µmol mol^{-1} for two rice (*Oryza sativa*) accessions. Each point is the mean (± SE) of four plants. The transition from low light to high light (50 to 1700 µmol m^{-2} s^{-1}) – i.e. the beginning of induction – begins at t = 0.

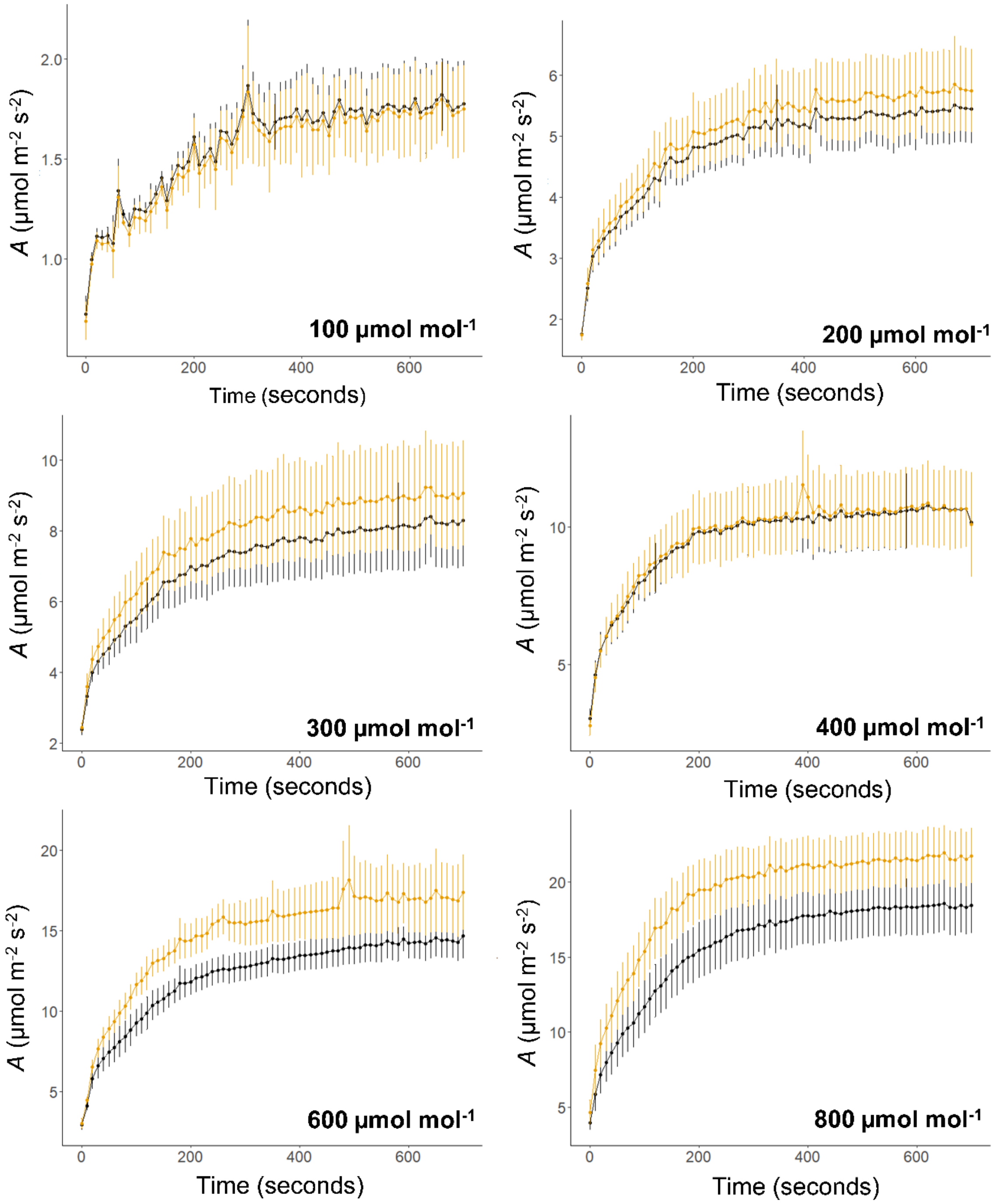

Fig. S4. The response of uncorrected leaf CO_2 assimilation (*A*) (black) and the response of leaf CO_2 assimilation corrected for stomatal limitation (A^*) (yellow) at different [CO_2]: 100, 200, 300, 400, 600, 800 (μmol mol^{-1}) in rice accession AUS 278. Each point is the mean (± SE) of 4 plants.

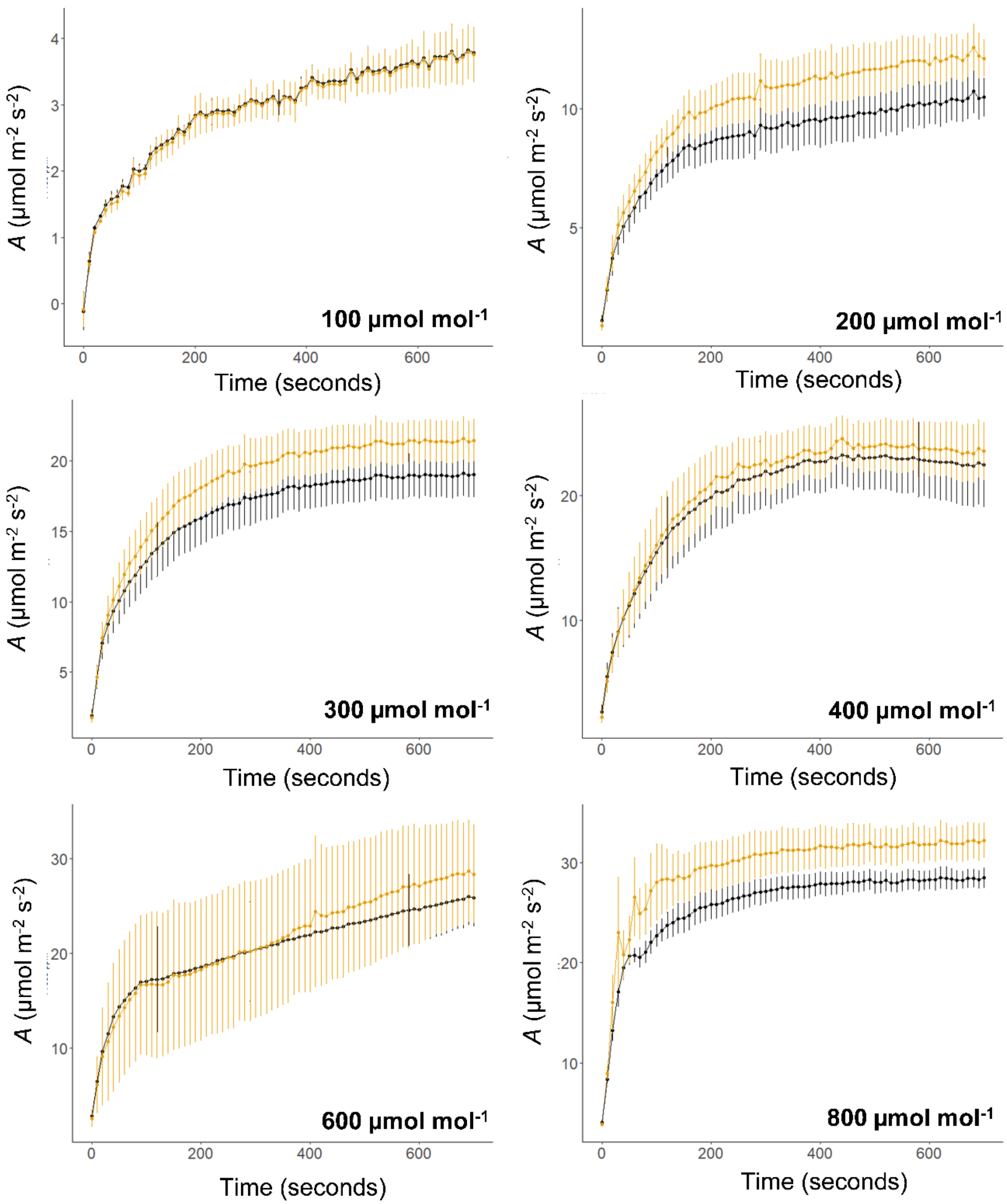

Fig. S5. The response of uncorrected leaf CO_2 assimilation (*A*) (black) and the response of leaf CO_2 assimilation corrected for stomatal limitation (A^*) (yellow) at different [CO_2]: 100, 200, 300, 400, 600, 800 (μmol mol^{-1}) in rice accession IR64-21. Each point is the mean (± SE) of 4 plants.

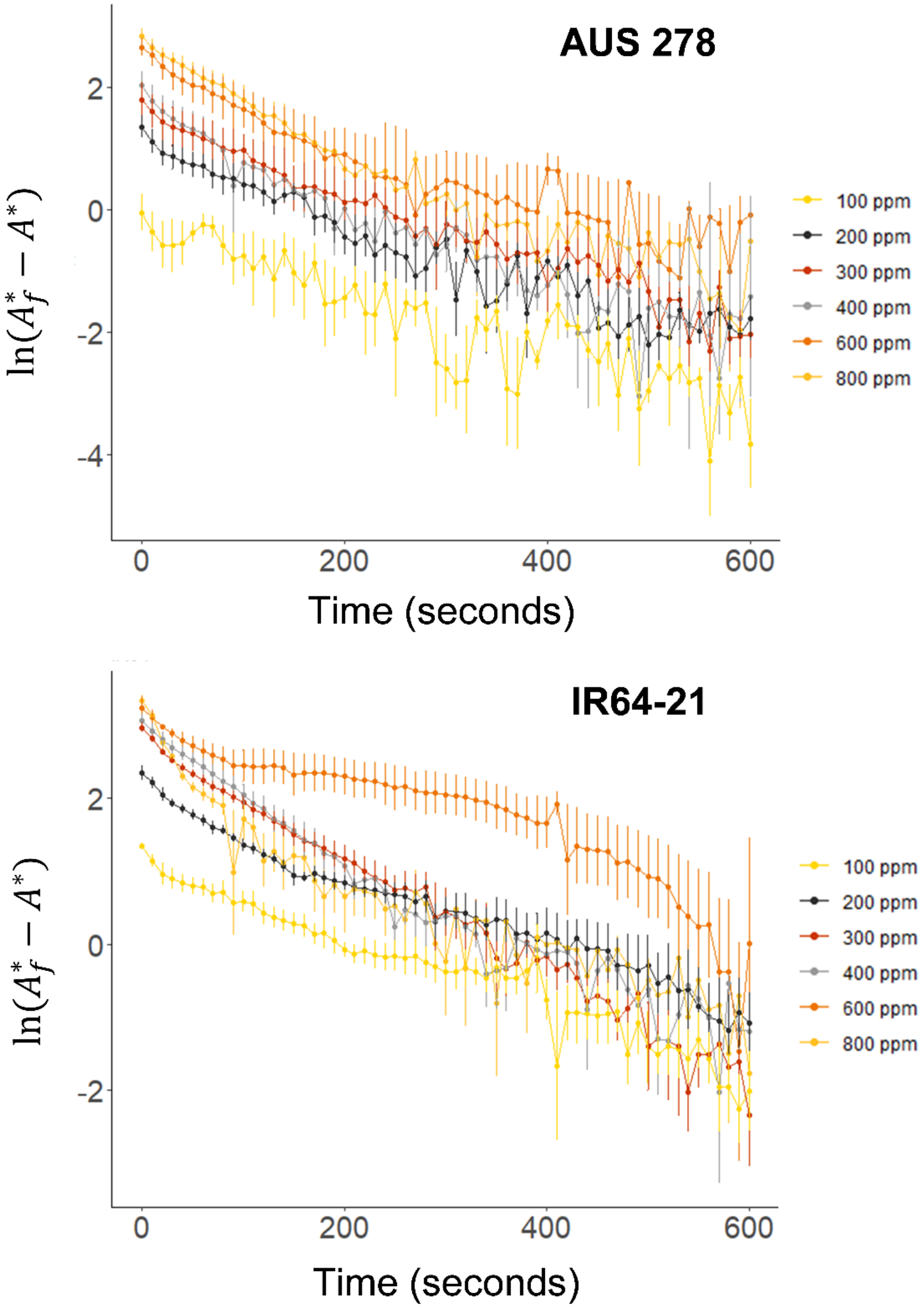

Fig. S6. The natural log of (A_f^*-A^*) over time, where A_f^* is CO_2 assimilation corrected for stomatal limitation at the end of photosynthetic induction and A^* is corrected CO_2 assimilation at a point in time (*t*) during induction. Each symbol represents a different [CO_2] at which photosynthetic induction was measured: 100, 200, 300, 400, 600, 800 µmol mol^{-1}. Each point is the mean of four plants ± SE (n = 4). Two rice accessions, AUS 278 and IR64-21, are presented.

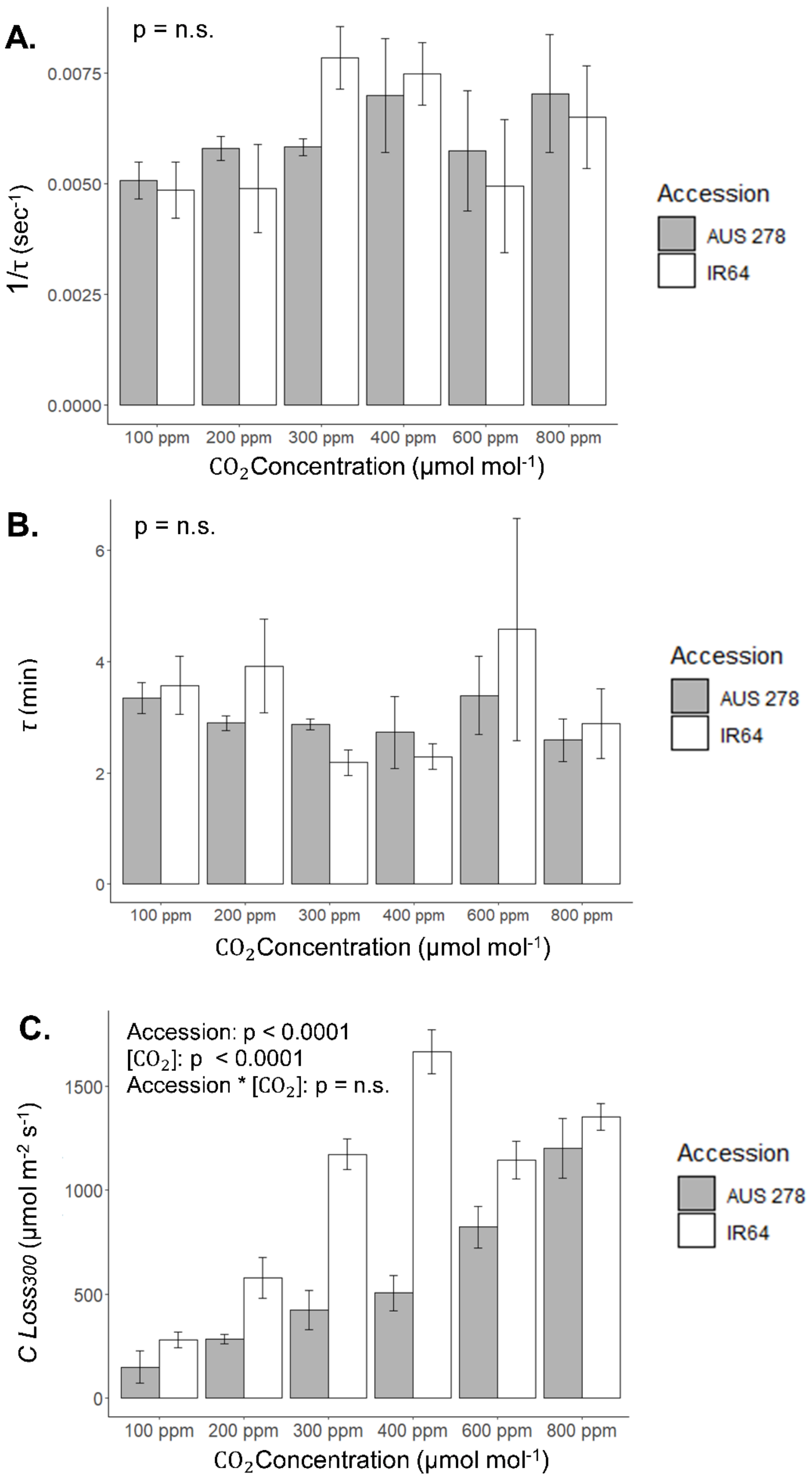

A.
p = n.s.
0.0075
0.0050
0.0025
0.0000
1/τ (sec-1)
100 ppm
200 ppm
300 ppm
400 ppm
600 ppm
800 ppm
CO2 Concentration (μmol mol-1)
Accession
AUS 278
IR64
B.
p = n.s.
6
4
2
0
τ (min)
100 ppm
200 ppm
300 ppm
400 ppm
600 ppm
800 ppm
CO2 Concentration (μmol mol-1)
Accession
AUS 278
IR64
C.
Accession: p < 0.0001
[CO2]: p < 0.0001
Accession * [CO2]: p = n.s.
1500
1000
500
0
C Loss300 (μmol m-2 s-1)
100 ppm
200 ppm
300 ppm
400 ppm
600 ppm
800 ppm
CO2 Concentration (μmol mol-1)
Accession
AUS 278
IR64

Fig. S7. Comparison between AUS 278 and IR64-21 at different [CO_2] concentrations: 100, 200, 300, 400 600, 800 µmol mol^{-1} for **3A.** The constant rate of Rubisco activation (1/τ), **3B.** The time to Rubisco activation in minutes (τ), and **3C.** forgone assimilation integrated over the first 300 seconds of photosynthetic induction ($C\ Loss_{300}$). Each bar is the mean (± SE) of 4 plants (n = 4).

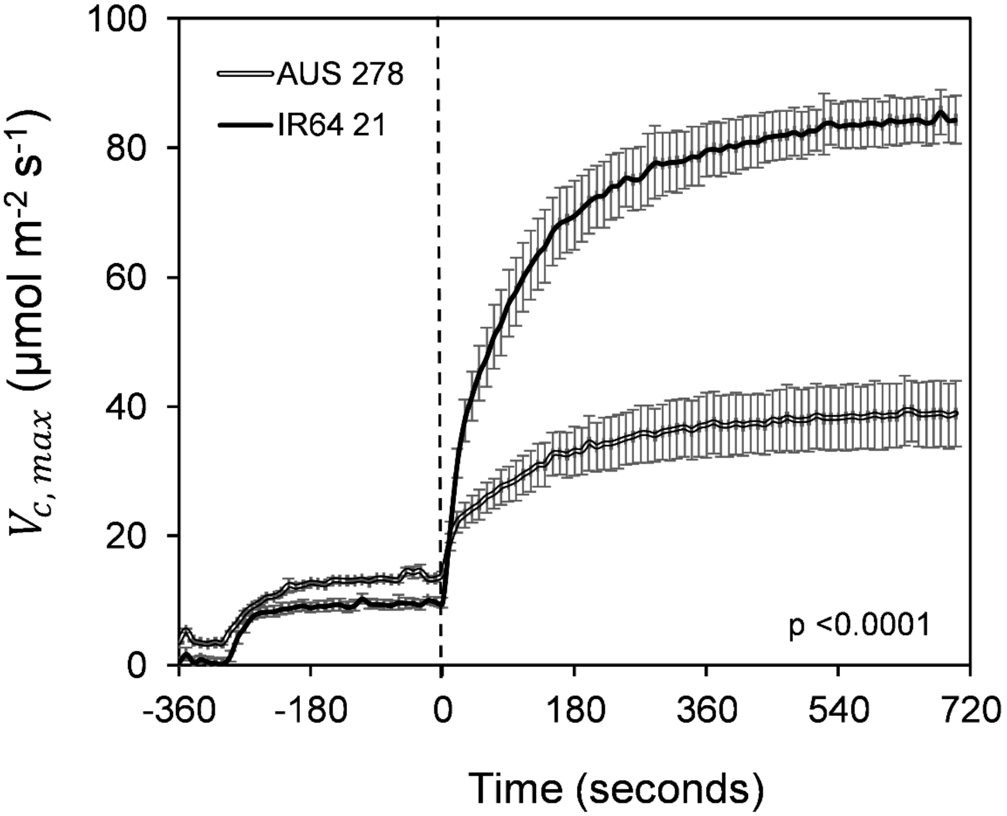

Fig. S8. The apparent maximum rate of carboxylation (*$V_{c,max}$*) with time through induction calculated from the response of leaf CO_2 uptake (*A*) to intercellular [CO_2] (*C_i*). Derived from inductions made at different [CO_2], as in Fig. 7. Each point represents the mean (± SE) of 4 individual plants for IR64-21 and AUS 278.

Table S1. Accessions selected from the 3000 Rice Genome Project (3K RGP) used to characterize natural genetic variation for photosynthetic induction in rice. The IRGC number, subpopulation, and country of origin are indicated.

**Accession #**	**Accession Name**	**IRGC Number**	**Subpopulation**	**Geographic Region**
**1**	AUS 278	IRGC 127182	Aus	Bangladesh
**2**	Dechangbyeo	IRGC 128275	Temperate *Japonica*	South Korea
**3**	Fei Zhao 12	IRGC 128291	Temperate *Japonica*	China
**4**	M 102	IRGC 125620	Tropical *Japonica*	United States
**5**	Malogbana	IRGC 128379	Admixed	Cote d'Ivoire
**6**	IR64-21	IRGC 135929	*Indica*	Philippines
